# Mechanical stretch promotes hypertrophic scar formation through mechanically activated cation channel Piezo1

**DOI:** 10.1038/s41419-021-03481-6

**Published:** 2021-03-01

**Authors:** Jiahao He, Bin Fang, Shengzhou Shan, Yun Xie, Chuandong Wang, Yifan Zhang, Xiaoling Zhang, Qingfeng Li

**Affiliations:** 1 Department of Plastic and Reconstructive Surgery, Shanghai Ninth People’s Hospital, Shanghai Jiao Tong University School of Medicine, 200011 Shanghai, China; 2 Department of Orthopedic Surgery, Xin Hua Hospital Affiliated to Shanghai Jiao Tong University School of Medicine (SJTUSM), 200092 Shanghai, China

**Keywords:** Cell signalling, Mechanisms of disease

## Abstract

Hypertrophic scar (HS) formation is a skin fibroproliferative disease that occurs following a cutaneous injury, leading to functional and cosmetic impairment. To date, few therapeutic treatments exhibit satisfactory outcomes. The mechanical force has been shown to be a key regulator of HS formation, but the underlying mechanism is not completely understood. The Piezo1 channel has been identified as a novel mechanically activated cation channel (MAC) and is reportedly capable of regulating force-mediated cellular biological behaviors. However, the mechanotransduction role of Piezo1 in HS formation has not been investigated. In this work, we found that Piezo1 was overexpressed in myofibroblasts of human and rat HS tissues. In vitro, cyclic mechanical stretch (CMS) increased Piezo1 expression and Piezo1-mediated calcium influx in human dermal fibroblasts (HDFs). In addition, Piezo1 activity promoted HDFs proliferation, motility, and differentiation in response to CMS. More importantly, intradermal injection of GsMTx4, a Piezo1-blocking peptide, protected rats from stretch-induced HS formation. Together, Piezo1 was shown to participate in HS formation and could be a novel target for the development of promising therapies for HS formation.

## Introduction

Hypertrophic scar (HS) formation is a skin fibroproliferative disease that occurs following a cutaneous injury, inducing severe functional and esthetic disability^1^. To date, numerous treatments have been established for inhibiting HS formation, but few of them showed satisfactory outcomes^2^. Therefore, further elucidation of molecular mechanisms of HS formation will contribute to developing novel treatments. Although numerous attempts have focused mainly on the traditional cytokine-mediated mechanisms involved in HS development^3^, more recent studies have demonstrated a key role for biomechanical cues in scarring^4,5^. Dermal fibroblasts, the end effectors of HS formation, have been identified as some of the key mechanosensitive cells in the skin^6^. Cutaneous trauma occurring in sites undergoing higher mechanical strain is more likely to induce HS formation, largely due to stretch resulting in fibroblasts to myofibroblasts differentiation, which is characterized by neo-expression of α-SMA and overproduction of extracellular matrix (ECM)^7^. Some important fibroblasts membrane mediators, including integrins^8^, stretch ion channels^9^, and G protein-coupled receptors (GPCRs)^10^ are reportedly capable of promoting fibrosis in response to mechanical signals. However, the understanding of the molecular mechanisms of dermal fibroblasts mechanosensation and mechanotransduction is still rudimentary. The dermal fibroblasts surface mechanosensors that directly detect and transduce scar tissue mechanics to modulate HS formation remain to be completely identified.

In 2010, Piezo proteins, including Piezo1 and Piezo2, were identified as the members of a novel class of mechanically activated cation channels (MACs)^11^. Piezo1 and Piezo2 are expressed in different cell types, and each modulates a different set of physiological processes^12^. Piezo2 plays an important role in neurosensory systems, such as proprioception^13^ and gentle touch sensation^14^. Although Piezo1 has not been shown to be involved in sensory functions, it is expressed in a wider range of tissues and implicated in multiple physiological functions^15–18^. Recently, research has demonstrated that Piezo1 actively interacts with the extracellular mechanical microenvironment to determine cell behaviors or diseases progression^19^. For example, Piezo1 plays an important role in detecting ECM elasticity and modulating the differentiation of neural stem cells^20^. In addition, Piezo1 relies on the mechanical input to modulate the tumor mechanical microenvironment and stimulate glioma growth^21^. It is noteworthy that tissue stiffness exacerbates the adult central nervous system progenitor cells aging properties through Piezo1 channel^22^. Therefore, Piezo1 might be a crucial factor in regulating mechanics-mediated cellular behaviors and diseases. Consistently, the predominant feature of scarring is the accumulation of ECM, which ultimately causes an increase in matrix stiffness^23^. A stiffer scar tissue establishes a “vicious feedback loop” that continuously stimulates fibroblasts overactivation and collagen production, further contributing to HS progression and contraction^24^. However, it is unknown whether the aberrant mechanical microenvironment in the scar interplays with the mechanosensory function of Piezo1 to regulate dermal fibroblasts activity, ultimately resulting in HS formation. Therefore, we postulated that the Piezo1 channel might be a key mediator involved in the induction of HS development by mechanical signals.

In this study, we demonstrated that the Piezo1 channel is overexpressed in human and rat HS tissues. We then used an in vitro Flexcell Tension system to explore changes in Piezo1 expression in HDFs under CMS. In addition, we examined the effects of Piezo1 activity on the fibroproliferative phenotypes of HDFs. Finally, GsMTx4, an inhibitor of the Piezo1 channel, was utilized to protect rats from stretch-induced HS formation.

## Materials and methods

### Patients samples

The human HS tissues and adjacent normal skin tissues used in this study were isolated from 9 people (Supplementary Table S1). All the samples obtained from Shanghai Ninth People’s Hospital with ethics approval from the local Human Research Ethics Committee of Shanghai Jiao Tong University School of Medicine in accordance with the principles of the Declaration of Helsinki.

### Isolation and culture of human dermal fibroblasts (HDFs)

The normal human skin tissues were cut into small pieces and placed in a 0.2% collagenase solution overnight at 4 °C. The next day, we used surgical scissors to excise the epidermis. The dermal skin was digested with 0.25% collagenase I solution (Sigma‐Aldrich, St Louis, MO, USA) at 37 °C for 6 h. After filtration, centrifugation, and resuspension, the HDFs were cultured in Dulbecco’s Modified Eagle Medium (DMEM) (Gibco Life Technologies, Grand Island, NY, USA) supplemented with 10% fetal bovine serum (FBS) (Gibco Life Technologies, Grand Island, NY, USA) and 1% penicillin/streptomycin (Gibco Life Technologies, Grand Island, NY, USA). The medium was changed every 3 days. Passage 3–6 HDFs were used for experiments.

### Mechanical stretch devices

Cyclic mechanical stretch (CMS) was applied to HDFs with an FX‐5000T™ Flexcell Tension Plus system (Flexcell International Corporation, Hillsborough, NC, USA). HDFs were cultured on 6-well BioFlex culture plates with flexible silicon membrane bottoms (Flexcell International Corporation, Hillsborough, NC, USA) at 2 × 10^5^ cells/ ml. CMS was applied to the stretch group in a sinusoidal pattern with a 10% amplitude at 0.5 Hz as previously reported^25^. Cells cultured in the same type of plate, but left static were incubated in the same incubator.

### Animal model

Adult male Sprague–Dawley rats aged 8 weeks were purchased from the Shanghai Slac Laboratory Animal (Slac, Shanghai, China). All procedures were approved by the Animal Care and Use Committee of Shanghai Jiao Tong University. A stretch-induced scarring model was generated based on a model established in our previous work^26^. In brief, a 6 × 6 mm incision was made on the tail of the rat. After hemostasis, the wounds were covered with dry sterile gauze. As soon as re-epithelialization finished, we fixed 2-cm diameter stainless steel rings to the side of the tail to stretch the wound site. Then, 12 rats were equally randomized into two groups: the PBS-treated group and the GsMTx4-treated group. We administered daily intradermal injections (300 µl) of PBS or GsMTx4 (5 µM) to the wound sites of the rats. The injection direction is parallel to the long axis of the scar. On day 14, all rats were sacrificed, and the scar tissues were surgically removed for experiments.

### RT-PCR

TRIzol reagent (Thermo Fisher Scientific, Waltham, MA, USA) was used to extract total RNA. The RNA was reverse transcribed into cDNA using RevertAid Reverse Transcriptase (Thermo Fisher Scientific, Waltham, MA, USA) according to the manufacturer’s instructions. Real-time PCR was performed with SYBR Premix EX Taq (Takara, Dalian, China) and LightCycler 480 System (Roche, Indianapolis, IN, USA). The primers used in our study were as follows: Piezo1 forward (5’-CCGCTCGTTTCCGAGTCAC-3’), Piezo1 reverse (5’-TGGTAGCAGTAGAGGCAGATG-3’), Piezo2 forward (5’-ACGACGATGCAAGGACATACG-3’), Piezo2 reverse (5’-GCTCACCAACGTGATGTGG-3’), β-actin forward (5’-CATGTACGTTGCTATCCAGGC-3’), β-actin reverse (5’-CTCCTTAATGTCACGCACGAT-3’). The sequences used were self-selected.

### Western blotting

All HDFs were lysed for 20 min with ice-cold RIPA lysis buffer. Ten micrograms of protein lysate (concentration determined by a bicinchoninic acid assay) was run on a 10% SDS-PAGE gel at 150 V for 1 h and then transferred to PVDF membranes. The membranes were blocked with 5% BSA at room temperature for 1 h. The membranes were incubated with primary antibodies against Piezo1 (Abcam, ab128245, 1:1000), collagen I (Abcam, ab34710, 1:500), collagen III (Abcam, ab7778, 1:500), fibronectin (Abcam, ab2413, 1:1000), α-SMA (CST, #19245, 1:1000), and GAPDH (CST, #5174, 1:1000) at 4 °C overnight. For Piezo1, the upper bands represent Piezo1 with a predicted mass of 287 kDa. For fibronectin, the upper bands represent fibronectin with a predicted mass of 262 kDa. For collagen I and collagen III, clear bands could be observed parallel to the 100 kDa size marker. For α-SMA and GAPDH, bands could be observed around 40 kDa size marker following manufacturer’s instruction. Quantitative analysis was performed on the immunoreactive bands with ImageJ software.

For Piezo1 peptide antigen competition experiments, we first dilute the necessary amount of antibody to the final volume needed (concentration: 1:1000, 1 µg/ml; final volume: 20 ml). Divide this equally into two tubes. In the first tube, labeled “Piezo1 antibody-control”. In the second tube, labeled “blocked”, add five times blocking peptide to the antibody by weight (50 µg total peptide in 10 ml buffer, concentration: 5 µg/ml). Incubate both tubes, with agitation, overnight at 4 °C. The next day, we prepared two identical samples (a western blot with two identical lanes, cut in half). Perform the staining protocol on the two identical samples, using the blocked antibody for one and the control of the other.

### Piezo1 inhibitor treatment

The Piezo1 inhibitor GsMTx4 (Alomone Labs, Jerusalem, Israel) was purchased and dissolved in PBS solution. According to a previous study, 5 µM GsMTx4 was used for all our experiments^27^.

### siRNA and transfection

For Piezo1 silencing, HDFs were transfected in 6-well plates with 100 nM Piezo1 siRNA by using Lipofectamine RNAiMAX reagent (Invitrogen, Carlsbad, CA, USA) according to the manufacturer’s protocol. The sequences were as follows: Piezo1-siRNA, 5’-AGAAGAAGAUCGUCAAGUATT-3’ (sense) and 5’-UACUUGACGAUCUUCUUCUTT-3’ (antisense), negative control (NC) siRNA, 5’-GUGAGCGUCUAUAUACCAUTT-3’ (sense) and 5’-AUGGUAUAUAGACGCUCACTT-3’ (antisense). The location of siRNA oligos within the Piezo1 mRNA sequence is “AGAAGAAGAUCGUCAAGUATT 6756–6774”. The sequences that we used were self-selected.

### Calcium imaging

HDFs were seeded in BioFlex culture plates and incubated overnight at 37 °C and 5% CO_2_. For Piezo1 silencing group, HDFs were pretreated with siRNAs for 72 h. The next day, the HDFs were loaded with Fluo-8-AM ester (5 μM; Abcam, Cambridge, UK) and simultaneously subjected to CMS (10%, 0.5 Hz) for 1 h. After CMS, the HDFs were washed with PBS 3 times and then rested for 20 min to allow complete processing of the AM ester by intracellular esterases. Fluorescence images of Ca^2+^ were taken with a Nikon Eclipse E800 microscope (Nikon, Melville, NY, USA). Fluorescence intensity measurements were analyzed using ImageJ software.

### Cell migration assay

Cell migration assays were performed using Transwell chambers (Corning, NY, USA). HDFs were seeded in DMEM without FBS in the upper chambers. The lower chambers were filled with DMEM with 10% FBS. After 24 h, the migrated HDFs were fixed and stained for 15 min in a 0.5% crystal violet solution. Images of migrated HDFs on the lower filters within three random fields were captured with a microscope.

### Apoptosis assay

The Annexin V Apoptosis Detection Kit (BD Biosciences, San Jose, CA, USA) was utilized to assess cellular apoptosis. HDFs were washed, centrifuged, and then resuspended in PBS buffer containing annexin V-FITC. The cells were incubated at room temperature for 15 min. The samples were analyzed with a Gallios flow cytometer (Beckman Coulter, Brea, CA, USA).

### Immunofluorescence

Cell samples were fixed in 4% paraformaldehyde in PBS for 15 min at room temperature. Tissue samples were fixed in 4% paraformaldehyde in PBS overnight at room temperature. The tissue samples were embedded, frozen, and sliced. Both the cell and tissue samples were then washed, permeabilized, and blocked. We used the following antibodies to investigate protein expression in these samples: anti-Piezo1 (Abcam, ab128245, 1:100), anti-Ki67 (CST, #9449, 1:400), anti-F-actin (Abcam, ab130935, 1:500), anti-α-SMA (CST, #48938, 1:200), Alexa Fluor 594 goat anti-rabbit secondary antibody (Jackson lab, 125369, 1:200), and Alexa Fluor 488 goat anti-mouse secondary antibody (Jackson lab, 133384, 1:200). Images were captured using a confocal microscope (Zeiss, Germany) or a Nikon Eclipse E800 microscope (Nikon, Melville, NY, USA).

### Histology and immunohistochemistry

Tissue samples were fixed in 4% paraformaldehyde in PBS, embedded and sliced. After deparaffinization and rehydration, the sections were stained with hematoxylin and eosin (H&E), Masson’s trichrome and picrosirius red (Solarbio, Beijing, China). We used the following antibodies to investigate protein expression in these samples by immunohistochemistry: anti-Piezo1 (Abcam, ab128245, 1:100) and anti-α-SMA (CST, #19245, 1:200) primary antibodies and an HRP-conjugated goat anti-rabbit secondary antibody (Jackson lab, 138729, 1:500). Mean optical density (MOD) measurements of the proteins were analyzed using ImageJ software.

### Collagen gel contraction assay

HDFs were resuspended in collagen (Shengyou, Hangzhou, China), and 1 ml of this suspension was plated in each well of a 12-well plate. The plates were incubated at 37 °C for 30 min to allow collagen gel polymerization. After collagen gel polymerization, 1 ml DMEM supplemented with 10% FBS was added to each well. Both the medium and gel did not contain GsMTx4. After 48 h, the gels were released from the wells and photographed.

### Statistical analysis

A two-tailed Student’s *t*-test was used for comparisons between two groups. One-way ANOVA was used for comparing multiple groups. *P* < 0.05 was considered to indicate a significant difference. At least three independent replicates were used for each experiment. The results are expressed as the means ± SD.

## Results

### Piezo1 expression is increased in human and rat HS tissues

**Fig. 1:**
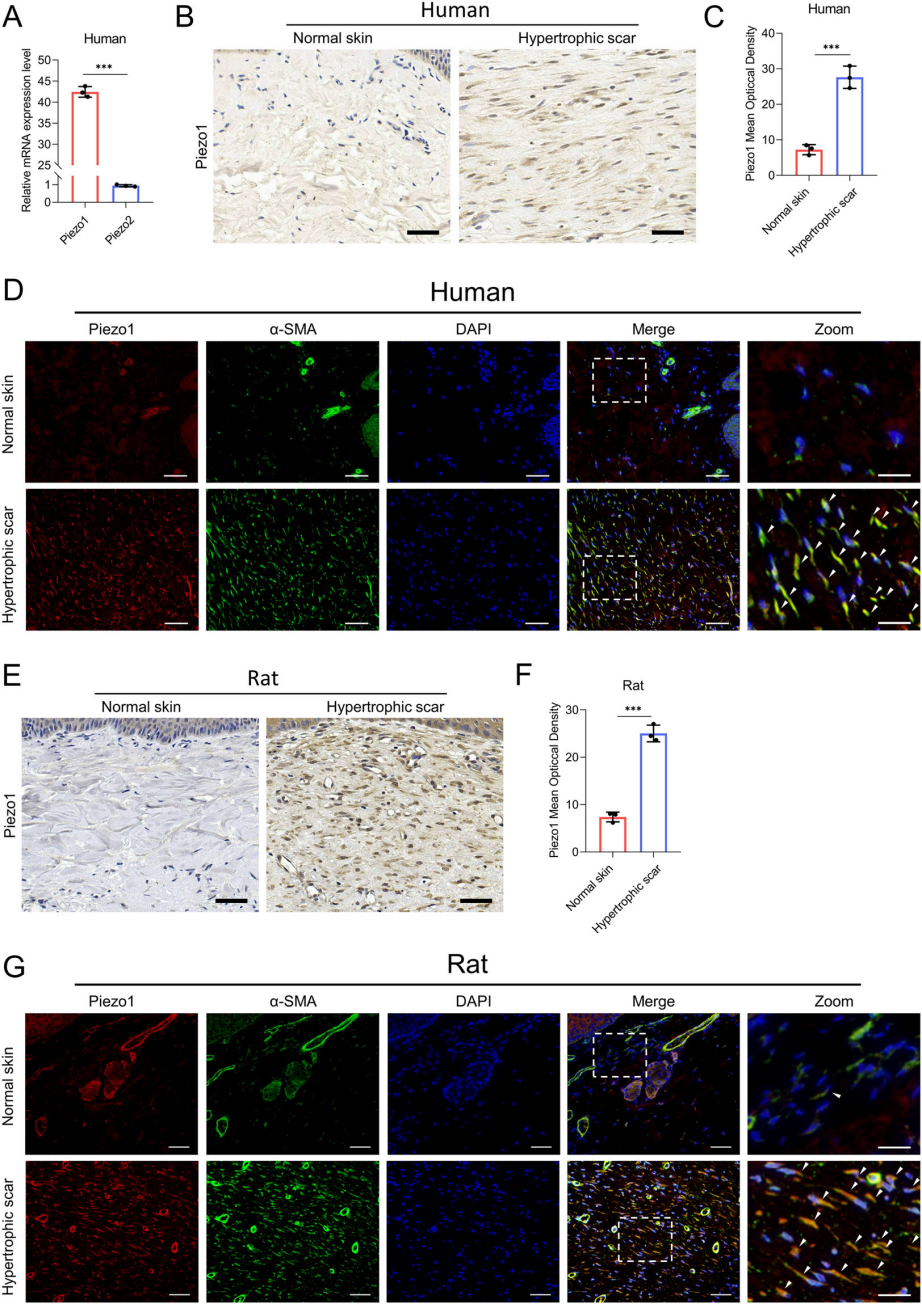
Piezo1 expression in normal skin and HS tissues. **A** The relative expression of Piezo1 and Piezo2 mRNA from HDFs was analyzed by real-time RT-PCR. **B**, **C** Images and quantitative analysis of Piezo1 in human normal skin and HS. (Scale bar = 50 μm). The basal epithelial layer was excluded from quantitation. **D** Images of immunofluorescence co-staining of Piezo1 and α-SMA in human normal skin and HS. Piezo1 are labeled in red and α-SMA in green. (Scale bar = 100 μm). Scale bars for the zoom images, 20 µm. **E**, **F** Images and quantitative analysis of immunohistochemistry staining of Piezo1 in rat normal skin and HS. (Scale bar = 50 μm). The basal epithelial layer was excluded from quantitation. **G** Images of immunofluorescence co-staining of Piezo1 and α-SMA in rat normal skin and HS. Piezo1 are labeled in red and α-SMA in green. (Scale bar = 100 μm). Scale bars for the zoom images, 20 µm. The arrowheads point to the fibroblasts. The results are expressed as the means with SD (*n* = 3). The *T*-test is used for all analysis. ***P < 0.005.

First, we analyzed the expression of Piezo1 and Piezo2 in HDFs. Quantitative reverse transcription PCR (RT-qPCR) showed that the mRNA level of Piezo1 was significantly higher than that of Piezo2 (Fig. 1A). We then studied the protein level of Piezo1 in normal skin and HS tissues from humans. The western blotting and immunofluorescence tested the specificity of Piezo1 antibody by siRNA-mediated knockdown of Piezo1 (Supplementary Fig. S1A–D). Immunohistochemical analysis confirmed an elevation in Piezo1 levels in human HS tissues (Fig. 1B, C). To further determine whether dermal fibroblasts show a high expression of Piezo1, we performed co-staining for α-SMA with Piezo1 to evaluate their co-localization in human HS tissues. As shown by immunofluorescence analysis, we observed that the expression of Piezo1 was correlated with the α-SMA level in HS tissues, suggesting that Piezo1 is highly expressed in myofibroblasts in human HS tissues (Fig. 1D). Furthermore, we studied Piezo1 expression in the rat tail model of stretch-induced HS. The immunohistochemical analysis revealed that Piezo1 is overexpressed throughout the rat HS dermis (Fig. 1E, F). We also performed co-staining for Piezo1 and α-SMA to investigate Piezo1 expression in rat dermal fibroblasts. The immunofluorescence assay revealed obvious co-localization of Piezo1 and α-SMA in rat HS tissues, similar to the phenomenon observed in human HS tissues (Fig. 1G). Taken together, these results confirm our hypothesis that Piezo1 might be associated with HS formation.

### CMS stimulates Piezo1 overexpression and Piezo1-dependent calcium influx in HDFs

**Fig. 2:**
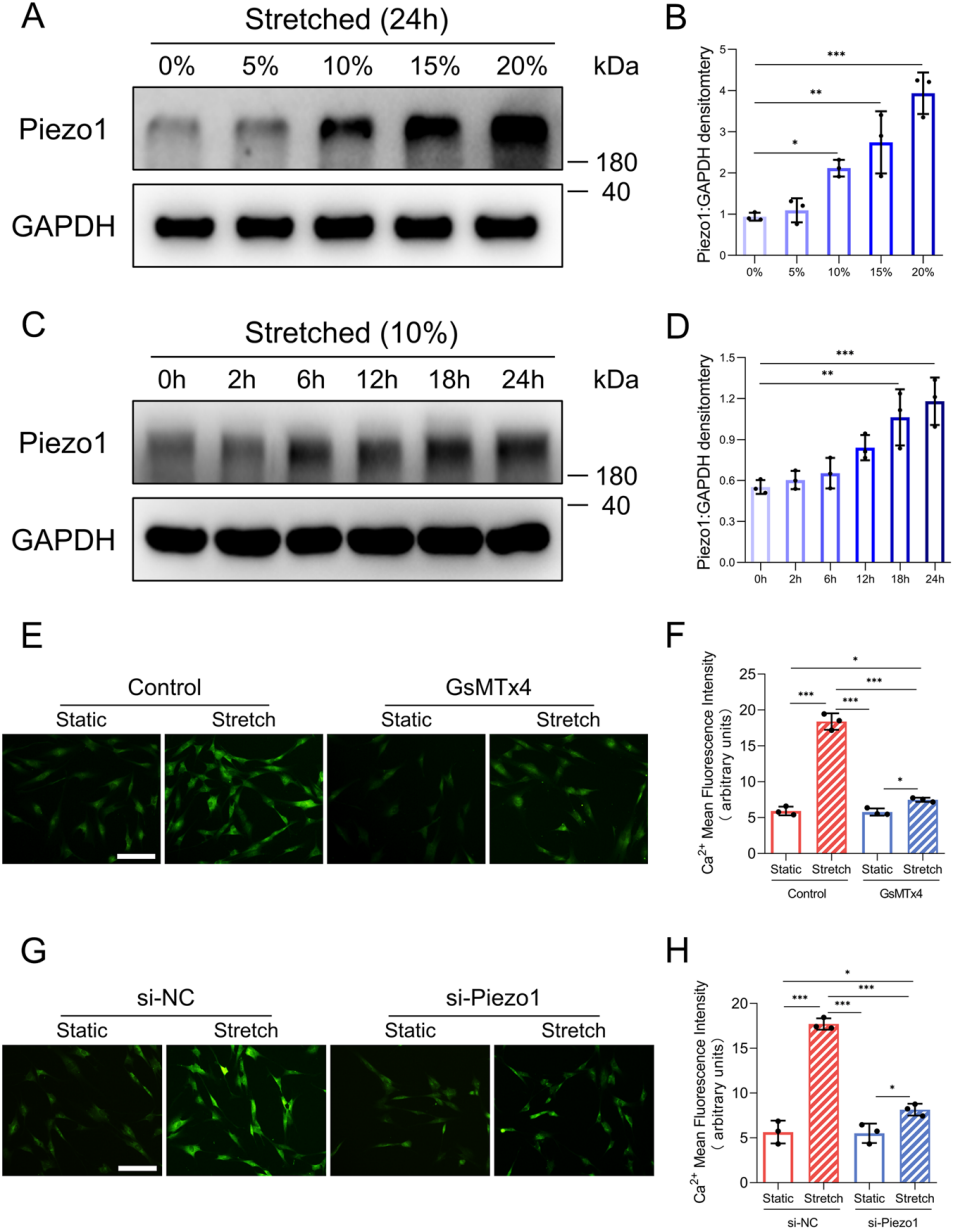
Piezo1 expression and Piezo1-dependent calcium influx in HDFs subjected to CMS. **A** Expression of Piezo1 in HDFs was determined by western blotting in a strength dependent manner. **B** Quantitative analysis of the expression level of Piezo1. **C** Expression of Piezo1 in HDFs was determined by western blotting in a time-dependent manner. **D** Quantitative analysis of the expression level of Piezo1. **E** Representative fluorescence images of intracellular Ca^2+^ in HDFs. (Scale bar: 100 μm). **F** Mean fluorescence intensity quantification of Ca^2+^ levels in HDFs. **G** Representative fluorescence images of intracellular Ca^2+^ in HDFs in the context of Piezo1 knockdown. (Scale bar: 100 μm). **H** Mean fluorescence intensity quantification of Ca^2+^ levels in HDFs in the context of Piezo1 knockdown. Ca^2+^ concentration assay in HDFs was performed in the condition of mechanical stretch (10%, 1 h) and GsMTx4 exposure (1 h). The results are expressed as the means with SD (*n* = 3). One-way ANOVA is used for all analysis. *P < 0.05, **P < 0.01, ***P < 0.005.

As shown in the in vivo results, Piezo1 is highly expressed in myofibroblasts of the HS. Previous studies have shown that mechanical force, especially matrix stiffness, could promote Piezo1 overexpression^21,22^. To investigate whether the increased matrix stiffness of HS tissues^28^ influences Piezo1 upregulation, we implemented a CMS model to mimic the increasing tissue stiffness during HS development^29^. We first examined the protein level of Piezo1 in HDFs exposed to stretch at various strengths (Fig. 2A, B). Notably, we found that Piezo1 expression was upregulated by the increased strength. In addition, Piezo1 expression also increased overtime when a 10% stretch strength was applied (Fig. 2C, D). These findings indicated that CMS dramatically increased Piezo1 expression in HDFs in a time-dependent and strength-dependent manner. Furthermore, because Piezo1 is a transmembrane ion channel that facilitates calcium entry in response to mechanical stretch, we tested whether Piezo1 activity affects calcium influx in HDFs. We used two different approaches to investigate this question by applying the Piezo1 antagonist GsMTx4^30^ or siRNA-mediated knockdown of Piezo1. The results showed that CMS promoted intracellular calcium concentrations in HDFs loaded with a Ca^2+^-sensitive fluorescence dye (Fluo-8). Importantly, GsMTx4 treatment or Piezo1 knockdown inhibited intracellular calcium accumulation in HDFs induced by CMS, suggesting that Piezo1 mediated calcium influx in HDFs exposed to CMS (Fig. 2E–H). In addition, CMS did not alter HDFs cell size (Supplementary Fig. S2A, B). Taken together, these results indicate that CMS may promote Piezo1 overexpression and Piezo1-mediated calcium influx in HDFs.

### Piezo1 activity promotes HDFs proliferation and motility

**Fig. 3:**
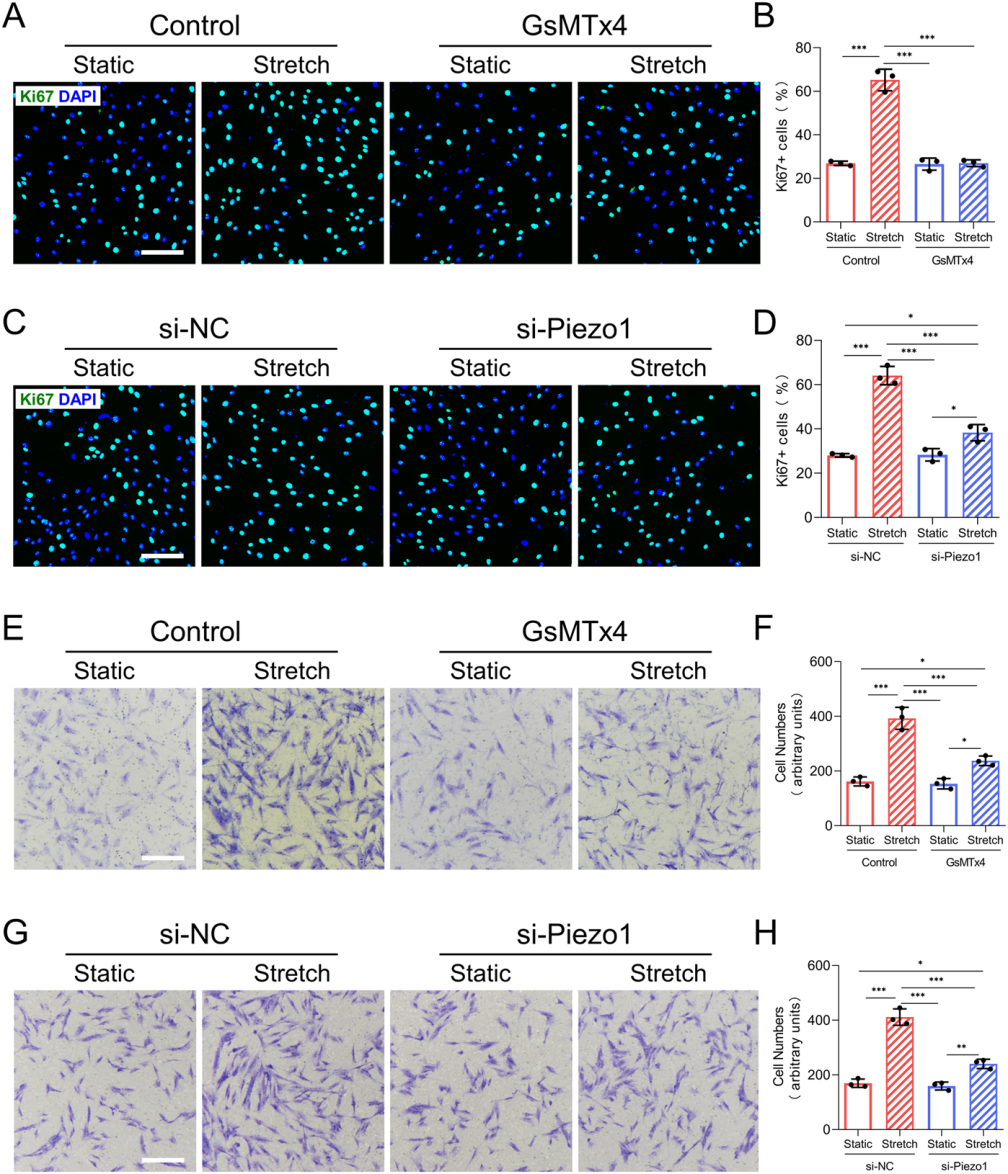
CMS induces HDFs proliferation and motility by activating Piezo1. **A** HDFs proliferative capacity was determined by Ki67 immunofluorescent staining in the context of GsMTx4 application. (Scale bar: 100 μm). **B** Quantitative analysis of the percentage of Ki67 positive cells. **C** HDFs proliferative capacity was determined by Ki67 immunofluorescent staining in the context of Piezo1 knockdown. (Scale bar: 100 μm). **D** Quantitative analysis of the percentage of Ki67 positive cells. **E** HDFs motility was detected by the Transwell migration assay in the context of GsMTx4 application. (Scale bar: 100 μm). **F** Quantitative analysis of the percentage of cell migration. **G** HDFs motility was detected by the Transwell migration assay in the context of Piezo1 knockdown. (Scale bar: 100 μm). **H** Quantitative analysis of the percentage of cell migration. Results are expressed as the means with SD (*n* = 3). One-way ANOVA is used for all analysis. *P < 0.05, **P < 0.01, ***P < 0.005.

Numerous studies have demonstrated that fibroblasts could sense mechanical force and then adopt a fibroproliferative phenotype, such as survival and motility^31,32^. To explore whether Piezo1 participates in mechanical force-induced fibroblasts proliferation, we performed Ki67 immunofluorescence staining to evaluate HDFs proliferative capacity. We found that CMS significantly increased the percentage of Ki67-positive cells compared to that in the static group, while GsMTx4 treatment or Piezo1 knockdown significantly inhibited stretch-induced HDFs proliferation (Fig. 3A–D). This indicated that Piezo1 might participate in stretch-induced HDFs proliferation. We next conducted a flow cytometry assay (Annexin V staining) to detect whether Piezo1 activity regulates cellular apoptosis in HDFs in vitro. The results showed that apoptotic cell populations were similar in all groups (Supplementary Fig. S3A–D). Furthermore, by using the Transwell migration assay, we found that CMS significantly increased the motility of HDFs. However, GsMTx4-treated or Piezo1-transfected HDFs exhibited poor migration ability in the context of CMS (Fig. 3E–H). Taken together, our findings reveal that Piezo1 might be involved in mechanical stretch-induced HDFs proliferation and motility.

### Piezo1 regulates HDFs differentiation in response to CMS

**Fig. 4:**
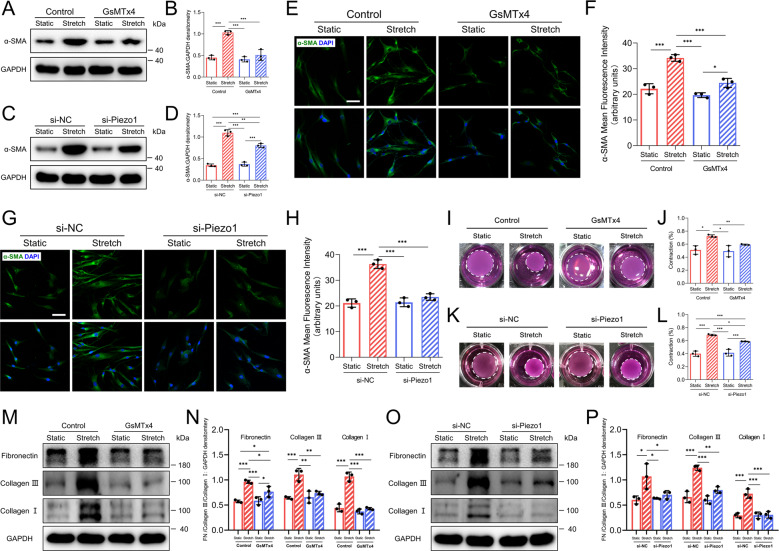
CMS promotes HDFs differentiation in a Piezo1-mediated mechanism. **A**, **B** Western blotting analysis of α-SMA in HDFs subjected (or not subjected) to mechanical stretch and treated (or not treated) with GsMTx4. **C**, **D** Western blotting analysis of α-SMA in HDFs subjected (or not subjected) to mechanical stretch and treated (or not treated) with Piezo1 knockdown. **E**, **F** Immunofluorescence analysis of α-SMA in HDFs subjected (or not subjected) to mechanical stretch and treated (or not treated) with GsMTx4. (Scale bar = 50 μm). **G**, **H** Immunofluorescence analysis of α-SMA in HDFs subjected (or not subjected) to mechanical stretch and treated (or not treated) with Piezo1 knockdown. (Scale bar = 50 μm). **I**, **J** Representative images and quantitative analysis of the collagen gel contraction assay in the context of GsMTx4 application. **K**, **L** Representative images and quantitative analysis of the collagen gel contraction assay in the context of Piezo1 knockdown. The “Contraction %” means the ratio of the area of each collagen gel to the area of the original well surface. **M**, **N** Western blotting analysis of fibronectin, collagen I, and collagen III in HDFs subjected (or not subjected) to mechanical stretch and treated (or not treated) with GsMTx4. **O**, **P** Western blotting analysis of fibronectin, collagen I, and collagen III in HDFs subjected (or not subjected) to mechanical stretch and treated (or not treated) with Piezo1 knockdown. For collagen I and fibronectin, two bands are included in densitometric quantitation. For collagen III, one band is included in densitometric quantitation. The results are expressed as the means with SD (*n* = 3). One-way ANOVA is used for all analysis. *P < 0.05, **P < 0.01, ***P < 0.005.

Fibroblasts to myofibroblasts differentiation is also an important factor directly corresponding to pathological tissue repair^33^. To determine the role of Piezo1 in HDFs differentiation, we first performed western blotting to assess the protein levels of α-SMA, a marker of activated fibroblasts, usually called myofibroblasts^34^. The results showed that stretched HDFs exhibited higher α-SMA levels than static HDFs. Meanwhile, GsMTx4 treatment or Piezo1 knockdown inhibited α-SMA overexpression when applied to HDFs cultured under CMS (Fig. 4A–D). In addition, immunofluorescence staining was conducted to further substantiate the changes in α-SMA, and the result was consistent with that obtained by western blotting (Fig. 4E–H). The α-SMA expression is directly proportional to the contractile force generated by myofibroblasts^35^. Thus, we used the collagen gel contraction assay to investigate scar tissue contracture resulting from myofibroblasts contraction. As expected, stretched HDFs showed a higher level of contractility than the non-stretched HDFs. Meanwhile, in the context of CMS, the contraction was less in GsMTx4-treated or Piezo1 knockdown HDFs. (Fig. 4I–L). Abundant collagen deposition is a dominant feature of scarring and results from excessive production of ECM-associated proteins by myofibroblasts. Therefore, we performed western blotting to investigate the role of Piezo1 in HDFs secretion. The results showed that collagen I, collagen III, and fibronectin expression was upregulated after CMS. As expected, inhibition or knockdown of Piezo1 significantly attenuated the secretion capacity of HDFs (Fig. 4M–P). Together, these results indicate that mechanical stretch-induced HDFs differentiation might be regulated by Piezo1 activity.

### Application of GsMTx4 attenuates excessive scarring in a stretch-induced rat tail model

**Fig. 5:**
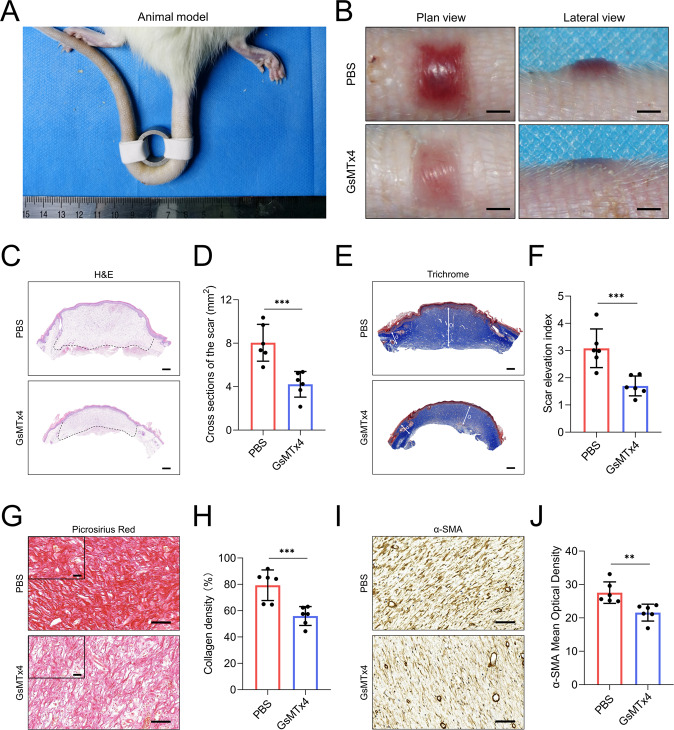
GsMTx4 attenuates excessive scarring in a stretch-induced rat tail model. **A** Animal modeling of stretch-induced scarring in rat tail. **B** Images of scar appearances and elevations in the PBS-treated group and GsMTx4-treated group (Scale bar = 2 mm). **C**, **D** Images and quantitative analysis of cross-sections of scar in PBS-treated group and GsMTx4-treated group. (Scale bar = 500 μm). **E**, **F** Images and quantitative analysis of SEI of scars in PBS-treated group and GsMTx4-treated group. (Scale bar = 500 μm). The long arrow ‘D’ represents the thickness of hypertrophic scar tissues while the short arrow ‘d’ represents the thickness of adjacent normal skin tissues. “scar elevation index” is defined as the D/d ratio. **G**, **H** Images and quantitative analysis of collagen density of scars in PBS-treated group and GsMTx4-treated group. (Scale bar = 50 μm). Scale bars for the zoom images, 50 µm. **I**, **J** Images and quantitative analysis of α-SMA expression in PBS-treated group and GsMTx4-treated group. (Scale bar = 50 μm). Arterioles were excluded from α-SMA quantitation. The results are expressed as the means with SD (*n* = 6). *T*-test is used for all analysis. **P < 0.01, ***P < 0.005.

**Fig. 6:**
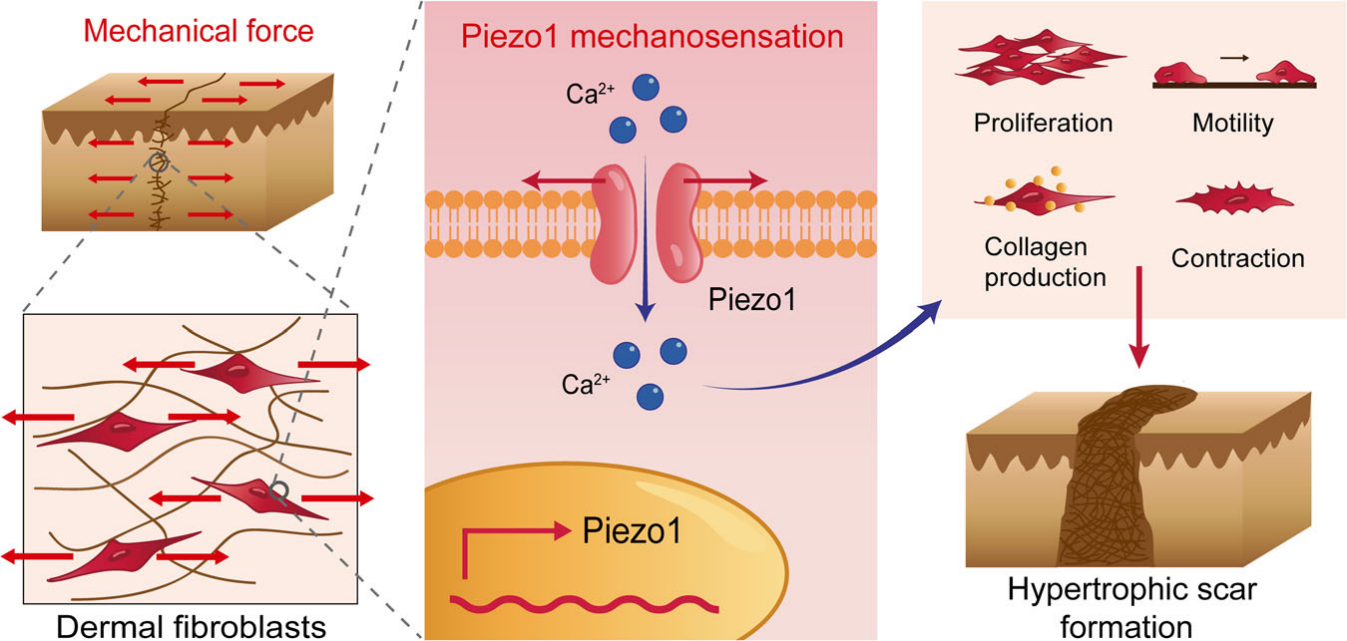
The schematic diagram of mechanical stretch promotes HS formation through Piezo1. During hypertrophic scar formation, mechanical force delivered from the extracellular matrix to Piezo1, which localizes at cell membranes of dermal fibroblasts. Then, mechanical force triggers Piezo1 overexpression and Piezo1-mediated calcium concentration in dermal fibroblasts. In addition, Piezo1 activity promotes proliferation, migration, and differentiation of dermal fibroblasts in response to mechanical force, subsequently contributing to mechanical force-induced hypertrophic scar formation.

To clarify the crucial role of Piezo1 in promoting mechanical stretch-induced HS, we examined the effects of GsMTx4 treatment in a model of stretch-induced scarring in rats^26^ (Fig. 5A). Macroscopic photographs of rat tail scars showed that GsMTx4 treatment decreased hyperemia and scar elevation (Fig. 5B). Histological analysis further demonstrated that the cross-section area and the scar elevation index (SEI) were reduced in GsMTx4-treated rats (Fig. 5C–F). In addition, we observed that collagen density was attenuated in the scars of GsMTx4-treated rats, as shown by picrosirius red staining (Fig. 5G, H). We further performed immunohistochemical staining to explore whether daily intradermal administration of GsMTx4 has an inhibitory effect on dermal fibroblasts function in vivo. As expected, α-SMA expression was lower in GsMTx4-treated rats than in PBS-treated rats (Fig. 5I, J). These findings demonstrate that continuous mechanical stretch applied to the wound site may induce HS formation through Piezo1 (Fig. 6).

## Discussion

The interplay between dermal fibroblasts and mechanical force regulates HS initiation and development, but the underlying mechanisms remain unclear^36^. The discovery of Piezo1, a novel MAC, led us to wonder whether Piezo1 participates in HS formation. In this study, we first revealed that Piezo1 is overexpressed in human and rat HS tissues. In addition, CMS triggers Piezo1 overexpression and Piezo1-mediated calcium concentration in HDFs in vitro. Furthermore, Piezo1 activity promotes proliferation, migration, and differentiation of dermal fibroblasts in response to mechanical stretch, subsequently contributing to stretch-induced HS formation. These findings demonstrate that mechanical stretch aggravates HS formation through a Piezo1-mediated mechanism.

The previous reports showed that Piezo1 and Piezo2 are expressed in different cell types^37^. Here, we found that HDFs exhibited high expression of Piezo1 and negligible levels of Piezo2, implying that Piezo1 predominantly expressed in HDFs (Fig. 1A). Furthermore, our results showed that Piezo1 was highly expressed in human and rat HS tissues, especially in myofibroblasts (Fig. 1B–G). Previous studies have demonstrated that a stiffer mechanical microenvironment significantly triggers Piezo1 overexpression^21,22^, suggesting that high Piezo1 expression might be associated with a higher level of mechanical force. Considering that HS tissues are stiffer than normal skin^28^, we hypothesized that Piezo1 overexpression in myofibroblasts might be associated with augmented matrix stiffness during HS formation. Therefore, we developed a CMS model to mimic the increased tissue stiffness during HS development as previously reported^29^. In vitro mechanical stretch assays indicated that CMS increased Piezo1 expression in HDFs in a time-dependent and strength-dependent manner, which further demonstrated that mechanical signals may upregulate Piezo1 expression in dermal fibroblasts (Fig. 2A–D). We also observed that CMS influenced Piezo1-dependent calcium concentrations in HDFs, which might be associated with dermal fibroblasts mechanotransduction (Fig. 2E–H). Previous studies have shown that Ca^2+^ is a universal key intracellular signaling element responsible for regulating various cellular processes^38^. It could modulate diverse biological characteristics of dermal fibroblasts such as proliferation, motility, and differentiation^39–41^. Thus, our findings implicate that the mechanical environment in HS might upregulate Piezo1 expression to enhance the mechanosensation and mechanotransduction capacities of dermal fibroblasts, leading to changes in the biological characteristics of dermal fibroblasts and subsequent HS development. Interestingly, a recent study has raised that Piezo1-mediated mechanosensation plays an essential role in potentiating pathogenic autoinflammation during pulmonary fibrosis^42^. This study further validates our hypothesis that Piezo1 may be associated with HS formation.

It is widely thought that dermal fibroblasts are the end effectors of HS formation^43,44^. A previous research emphasized that mechanical loading initiates HS formation through activation of fibroblasts survival pathways^31^. Previous studies have also illustrated that Piezo1 mediates mechanical force-induced proliferation of many cell types, including mouse embryonic stem cells^45^, human progenitors^46^, and osteoblastic cells^47^. In this study, our data showed that CMS promotes HDFs proliferation in a Piezo1-mediated manner (Fig. 3A–D). But there were no significant differences in the percentage of apoptotic cells among all groups (Supplementary Fig S3), which is consistent with a previous report indicating that survival pathway activation is not a major factor in HS formation^48^. In addition, fibroblasts migration into the wound clot is also an essential process of HS formation^49^. Previous reports have shown that Piezo1 is required for mechanical force-induced migration of CHO cells^50^, gastric cancer cells^51^, and epithelial cells^52^. Our in vitro motility assay also suggested that Piezo1 was necessary for stretch-induced HDFs migration (Fig. 3E–H). Furthermore, fibroblasts to myofibroblasts differentiation is another important factor directly associated with HS formation^53,54^. A recent study has shown that Piezo1 plays a key role during myofibroblast–fibroblast cross-talk during fibrosis expansion^55^. We observed that the effects of stretch-induced myofibroblasts differentiation were inhibited by treating HDFs with GsMTx4 or Piezo1 knockdown, indicating that myofibroblasts differentiation following mechanical stretch might require Piezo1 (Fig. 4). Taken together, our findings demonstrated that mechanical stretch promotes dermal fibroblasts proliferation, motility, and differentiation through Piezo1, which may ultimately lead to the formation of HS.

Numerous methods, such as lasers, external medicine, and corticosteroid injections, are utilized for HS treatment, but they seldom exhibit satisfactory outcomes^56^. It is noteworthy that several therapeutic approaches targeting the physical environment of the HS, including paper tape^57^, silicone sheeting^58^, and Botulinum toxin type A^59^, have shown some success in mitigating HS formation through mechanical offloading of the HS environment. Our research has emphasized that Piezo1-mediated mechanotransduction is critical for HDFs to adopt fibroproliferative phenotypes in vitro. Thus, the application of GsMTx4, which targets Piezo1, might potentially influence HS formation. Our in vivo study showed that GsMTx4 treatment effectively protects rats from stretch-induced HS formation (Fig. 5). Together, our results demonstrate that inhibition of Piezo1 might be a novel approach to alleviate HS development. Some studies have validated Piezo1 potential as a novel therapeutic target^60^. Genetic studies of humans and mice have unequivocally confirmed that Piezo1 is a novel therapeutic target for malaria infection and stomatocytosis^61,62^. The cryo-electron microscopy structure of Piezo1 and the identification of a Piezo1 agonist demonstrate the drugability of Piezo1 channel^63,64^. Piezo1 also exhibits therapeutic potential for aging and cancer immunotherapy^22,65^. In this study, we utilized GsMTx4 to silence Piezo1 as a means of “deceiving” dermal fibroblasts and thereby caused these cells to lose the ability to sense and transduce mechanical signals, effectively uncoupling mechanical stretch from HS formation. However, GsMTx4 is not known as a specific inhibitor for Piezo1, which is the limitation of this study. Therefore, a myofibroblast-specific Piezo1-deficient mouse mediated loss of function studies in the model of hypertrophic scar formation could be much more convincing to confirm our hypothesis.

The decreased biomechanical function of scars highlights the need for clinical therapies to improve the scar tensile strength^66^. It is reported that very few effective therapies exist which inhibit scar formation while maintaining scar tensile strength^67^. Ward et al. revealed that subcutaneous injection of GsMTx4 improved muscle strength of mice^68^. Thus, we wonder that whether GsMTx4 treatment could influence the tensile strength of the healed scar. Compared with healthy skin, scars have decreased tensile strength due to alteration in the architecture of fibrillar collagen^69^. We conducted picrosirius red staining and observed that collagen bundles were both irregular in the scars after treatment with or without GsMTx4 (Fig. 5G). Therefore, at this time, we could not draw the conclusion that GsMTx4 treatment has an influence on the tensile strength of the healed scar. Direct measurement of scar tensile strength using a microtester could be much more convincing^70^.

As for the proposed mechanism in Piezo1-mediated hypertrophic scar formation, our findings indicate that mechanical stretch might regulate scar formation through Piezo1-mediated Ca^2+^ pathways. Some studies identified several mechanosensitive molecules in the skin that influence scar formation, such as FAK, ERK, and Yap, which are also downstream effectors of Piezo1 channel^4,20,21,71^. Therefore, these might be the potential mechanism in Piezo1-mediated hypertrophic scar formation. It will be of great interest to further discover how Piezo1 activity contributes to hypertrophic scar formation.

In conclusion, our results confirm that Piezo1 activation may mediate HS formation. We explored how the negative response to mechanical stretch can be attenuated by mitigating the mechanosensory function of dermal fibroblasts—namely, by administering GsMTx4 or Piezo1-siRNA to inhibit Piezo1 activation. Our findings also imply that the application of the Piezo1-blocking peptide GsMTx4 may have potential as a therapeutic strategy for HS. More broadly, our findings raise the possibility that Piezo1 might be a general factor driving diseases that the aberrant mechanical force involved.

## Supplementary information

Supplementary Figure Legends

Supplementary Figure S1

Supplementary Figure S2

Supplementary Figure S3

Supplementary Figure S4

Supplementary Table
