# Supplementary material for: Mechanical stretch promotes hypertrophic scar formation through mechanically activated cation channel Piezo1
Source: Cell Death Dis. 2021 Mar 1;12(3):226. doi: 10.1038/s41419-021-03481-6 (PMC7921104; doi:10.1038/s41419-021-03481-6)
Supplement: Supplementary file 1 — Supplementary Figure Legends [file 41419_2021_3481_MOESM1_ESM.docx]

**Supplementary Figure legends**

**Supplementary Figure S1: siRNA mediated Piezo1 knockdown in HDFs.** (A) The efficiency of Piezo1 knockdown were validated through western blot analysis. (B) Quantitative analysis of Piezo1 protein level in HDFs. (C) The efficiency of Piezo1 knockdown were validated through immunofluorescence analysis. (Scale bar = 10 μm). (D) Mean fluorescence intensity quantification of Piezo1 protein level in HDFs. The results are expressed as the means with SD (n = 3). *T*-test is used for all analysis. **P < 0.01, ***P < 0.005.

**Supplementary Figure S2: Cyclic mechanical stretch (10%, 1h) does not affect HDFs size.** (A) The area of HDFs were determined by F-actin immunofluorescence. (Scale bar = 100 μm). (B) Quantitative analysis of HDFs area. The results are expressed as the means with SD (n = 3). *T*-test is used for analysis. ns: no significance.

**Supplementary Figure S3: Piezo1 activity did not affect HDFs apoptosis.**

(A) Apoptosis of HDFs was detected by flow cytometry assay in the context of GsMTx4 application. (B) Quantitative analysis of the apoptotic cell population. (C) Apoptosis of HDFs was detected by flow cytometry assay in the context of Piezo1 knockdown. (D) Quantitative analysis of the apoptotic cell population. The results are expressed as the means with SD (n = 3). One-way ANOVA is used for analysis.

**Supplementary Figure S4: Uncropped gel blots with size marker indication.**

(A) Uncropped gel blots with size marker indications for Figure 2A (containing gel blot for Piezo1 peptide competition). (B) Uncropped gel blots with size marker indications for Figure 2C (containing gel blot for Piezo1 peptide competition). The concentration of peptide used for competition experiments is 5 µg/ml. (C) Uncropped gel blots with size marker indications for Figure 4G. (D) Uncropped gel blots with size marker indications for Figure 4H. (E) Uncropped gel blots with size marker indications for Supplementary Figure S1A.
