# Supplementary figures and images for: Mechanical stretch promotes hypertrophic scar formation through mechanically activated cation channel Piezo1
Source: Cell Death Dis. 2021 Mar 1;12(3):226. doi: 10.1038/s41419-021-03481-6 (PMC7921104; doi:10.1038/s41419-021-03481-6)

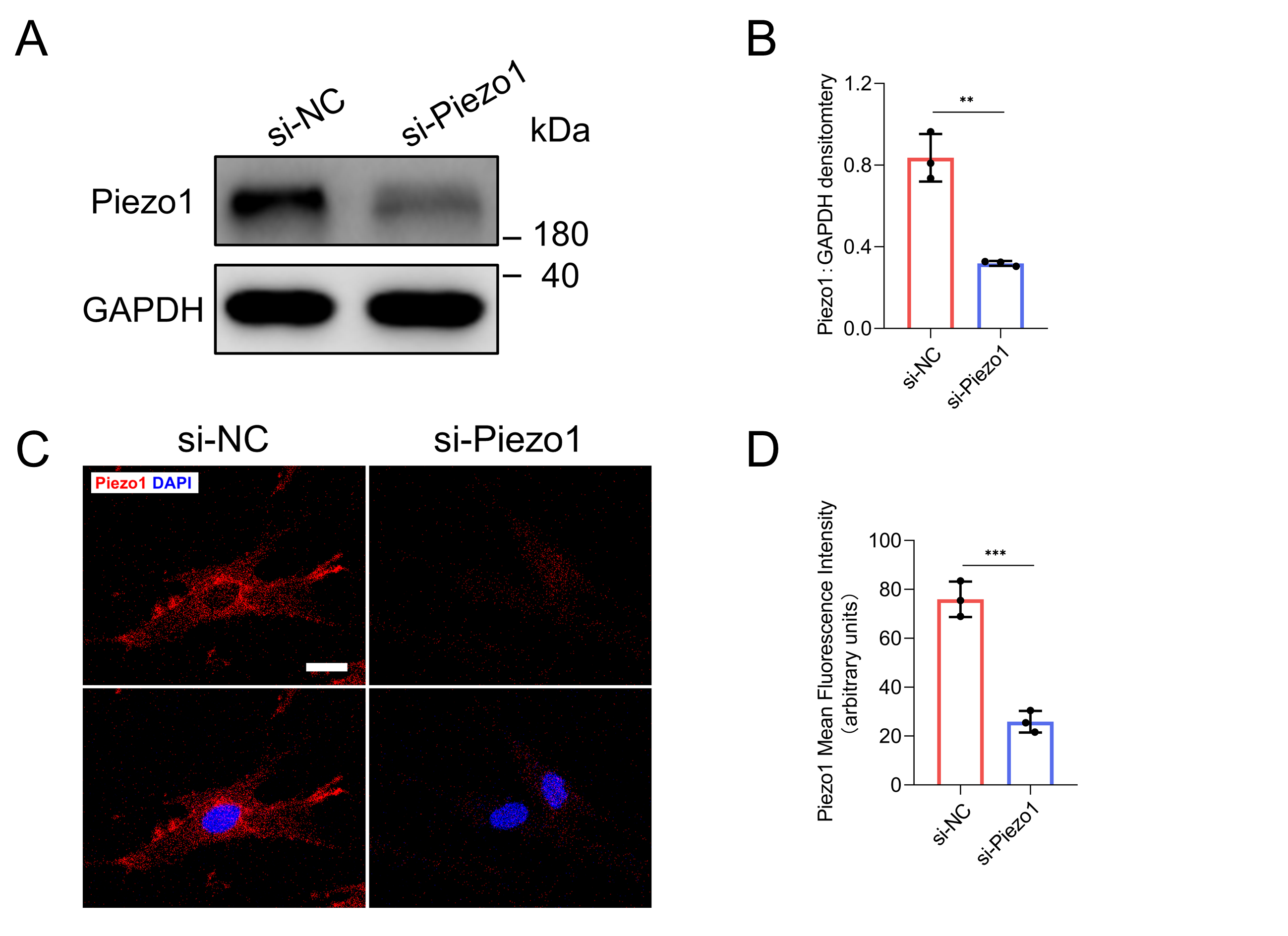

Supplement: Supplementary file 2 — Supplementary Figure S1 [file 41419_2021_3481_MOESM2_ESM.tif]

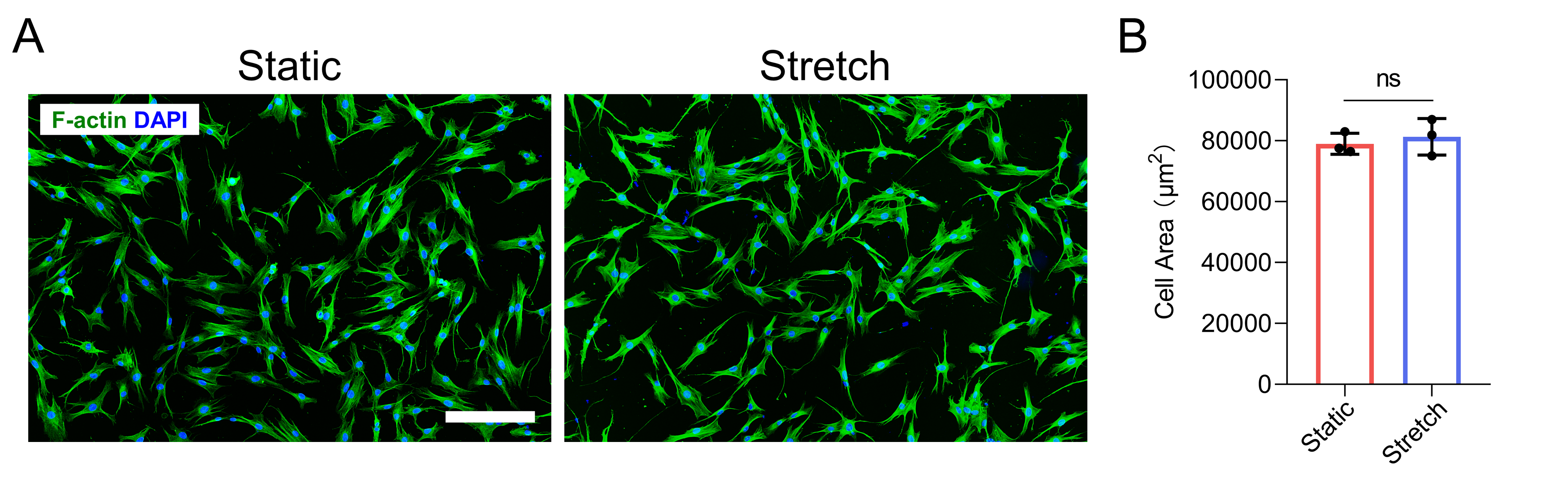

Supplement: Supplementary file 3 — Supplementary Figure S2 [file 41419_2021_3481_MOESM3_ESM.tif]

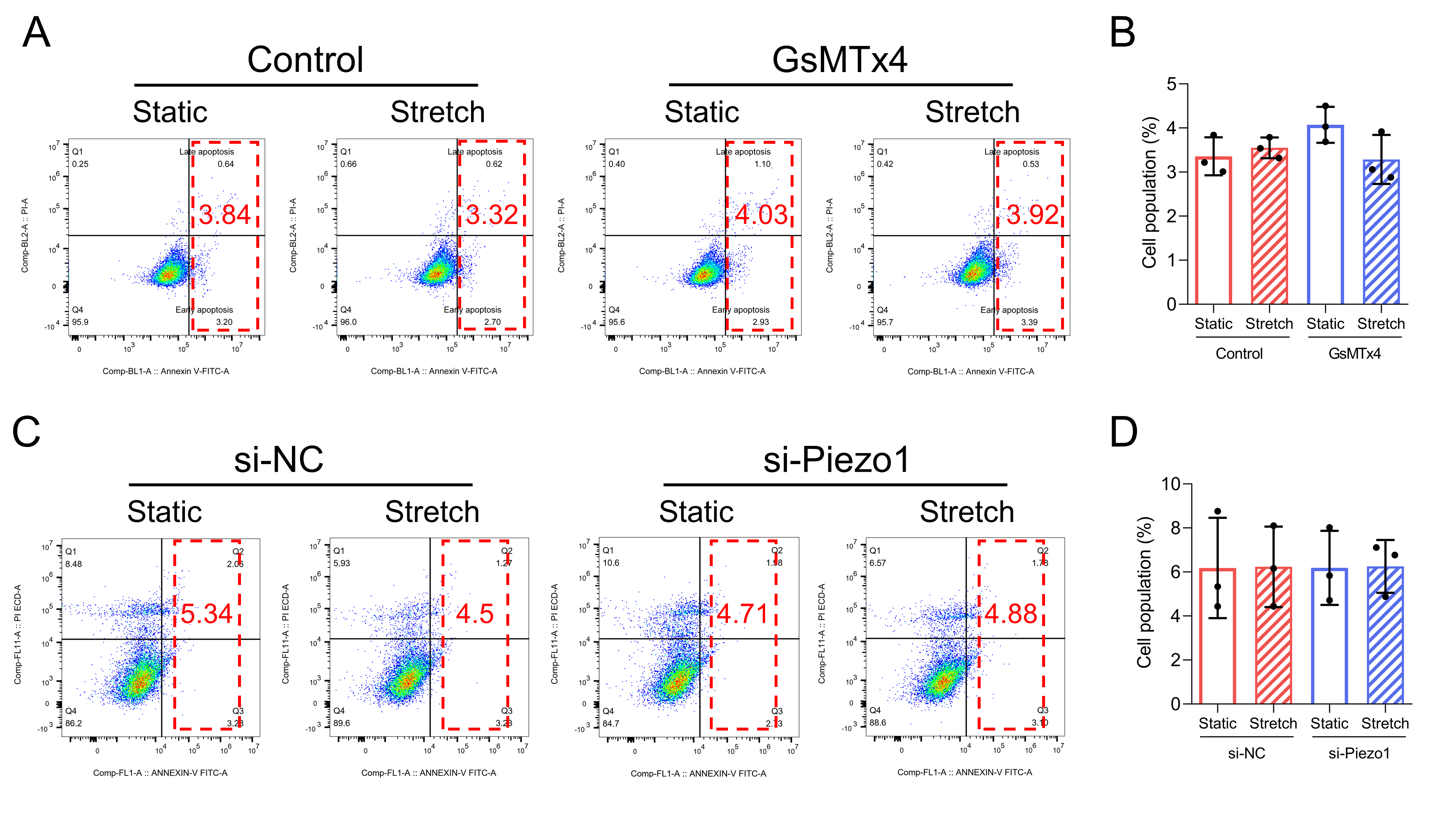

Supplement: Supplementary file 4 — Supplementary Figure S3 [file 41419_2021_3481_MOESM4_ESM.tif]

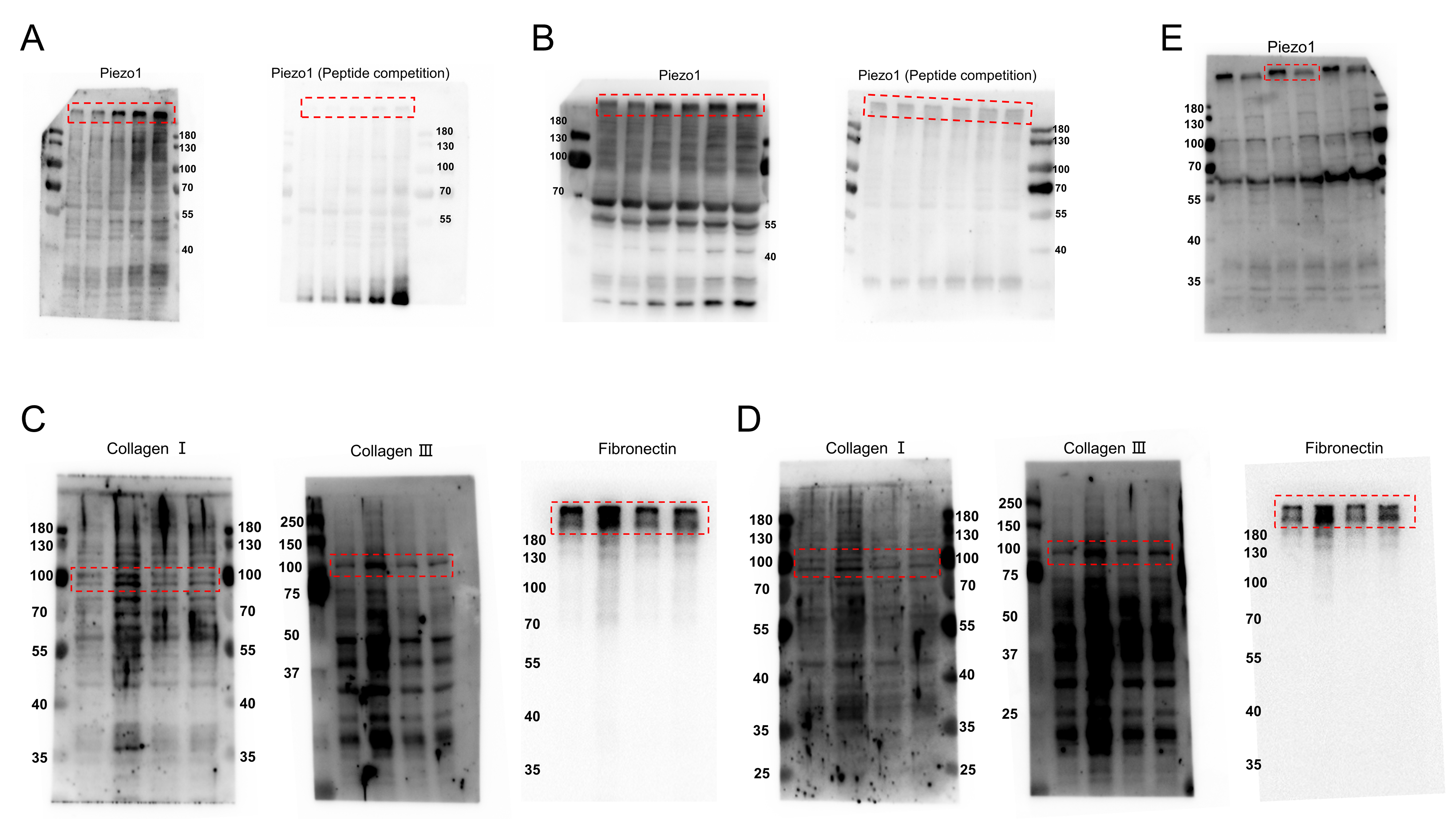

Supplement: Supplementary file 5 — Supplementary Figure S4 [file 41419_2021_3481_MOESM5_ESM.tif]
