# Supplementary material for: Mechanical stretch promotes hypertrophic scar formation through mechanically activated cation channel Piezo1
Source: Cell Death Dis. 2021 Mar 1;12(3):226. doi: 10.1038/s41419-021-03481-6 (PMC7921104; doi:10.1038/s41419-021-03481-6)
Supplement: Supplementary file 6 — Supplementary Table [file 41419_2021_3481_MOESM6_ESM.docx]

1. **Supplementary Table S1. Patients information**

| Patients Sex Age (years) Scar localization | | | |
| --- | --- | --- | --- |
| 1 | Female | 11 | Chest |
| 2 | Female | 33 | Belly |
| 3 | Male | 29 | Chin |
| 4 | Male | 5 | Arm |
| 5 | Male | 25 | Cheek |
| 6 | Male | 17 | Chin |
| 7 | Female | 9 | Cheek |
| 8 | Female | 14 | Cheek |
| 9 | Male | 21 | Chin |
